# A review and synthesis of frameworks for engagement in health research to identify concepts of knowledge user engagement

**DOI:** 10.1186/s12874-019-0838-1

**Published:** 2019-11-21

**Authors:** Janet E. Jull, Laurie Davidson, Rachel Dungan, Tram Nguyen, Krista P. Woodward, Ian D. Graham

**Affiliations:** 1 School of Rehabilitation Therapy, Faculty of Health Sciences, Queen’s University, 31 George Street, Louise D. Acton Building, Kingston, Ontario Canada; 2 Ottawa Hospital Research Institute, 501 Smyth Road, Ottawa, Ontario Canada; 3 Patient-Centered Outcomes Research Institute (PCORI), 1828 L Street, NW, Washington D.C., 20008 USA; 4 Clinical Epidemiology Program, Ottawa Hospital Research Institute, 501 Smyth Road, Ottawa, Ontario Canada; 5 CanChild Centre for Childhood Disability Research, Faculty of Health Sciences, McMaster University, Hamilton, Ontario Canada; 6 Public and Patient Engagement Department, Patient-Centered Outcomes Research Institute (PCORI), 1828 L Street, NW, Washington D.C., 20008 USA; 7 Department of Epidemiology and Community Medicine, University of Ottawa, Ottawa, Ontario Canada

**Keywords:** Engagement, Collaboration, Co-production, Model, Framework, Knowledge, Research, Health system, Integrated knowledge translation

## Abstract

**Background:**

Engaging those who influence, administer and/or who are active users (“knowledge users”) of health care systems, as co-producers of health research, can help to ensure that research products will better address real world needs. Our aim was to identify and review frameworks of knowledge user engagement in health research in a systematic manner, and to describe the concepts comprising these frameworks.

**Methods:**

An international team sharing a common interest in knowledge user engagement in health research used a consensus-building process to: 1) agree upon criteria to identify articles, 2) screen articles to identify existing frameworks, 3) extract, analyze data, and 4) synthesize and report the concepts of knowledge user engagement described in health research frameworks. We utilized the Patient Centered Outcomes Research Institute Engagement in Health Research Literature Explorer (PCORI Explorer) as a source of articles related to engagement in health research. The search includes articles from May 1995 to December 2017.

**Results:**

We identified 54 articles about frameworks for knowledge user engagement in health research and report on 15 concepts. The average number of concepts reported in the 54 articles is *n* = 7, and ranges from *n* = 1 to *n* = 13 concepts. The most commonly reported concepts are: knowledge user - prepare, support (*n* = 44), relational process (*n* = 39), research agenda (*n* = 38). The least commonly reported concepts are: methodology (*n* = 8), methods (*n* = 10) and analysis (*n* = 18). In a comparison of articles that report how research was done (*n* = 26) versus how research should be done (*n* = 28), articles about how research was done report concepts more often and have a higher average number of concepts (*n* = 8 of 15) in comparison to articles about how research should be done (*n* = 6 of 15). The exception is the concept “evaluate” and that is more often reported in articles that describe how research should be done.

**Conclusions:**

We propose that research teams 1) consider engagement with the 15 concepts as fluid, and 2) consider a form of partnered negotiation that takes place through all phases of research to identify and use concepts appropriate to their team needs. There is a need for further work to understand concepts for knowledge user engagement.

## Background

In health research, there is growing support for the engagement of patients, caregivers, clinicians, managers, decision makers and other healthcare systems users as co-producers of evidence. The participation of researchers with those who influence, administer and/or who are active users of healthcare systems create opportunities for processes of co-production of research evidence and that is a form of knowledge. Collaboration between researcher and health care systems users can take many forms, and is often referred to using different terminology, for example, integrated knowledge translation [1], collaborative research, co-production of knowledge [2], and engaged scholarship [3]. In all cases, however, there is a common aim: to produce and apply knowledge to address real-world needs. The resulting knowledge is more likely to be useful and useable and therefore more likely to be applied in policy and practice [3, 4]. We define "engagement" in research as an arrangement in the governance of the research process with those who influence, administer and/or who are active users of healthcare systems, and that leads to co-production of knowledge (beyond being a research participant) [5, 6].

Engagement in research disrupts the notion of research as the sole domain of academic researchers. The expansion of research roles invite many diverse forms of expertise into an involved and intentional process of knowledge production. We adopt and adapt the definitions developed by Parry et al. (2015) to describe the roles of those engaged in research that leads to knowledge production. The aim of engagement in research is to integrate the views and values of "knowledge users", meaning those who are actively involved in the knowledge production process of a study and who may benefit or be otherwise affected by the research [7]. We expand the definition of knowledge user, and include those who may not be members of the mainstream academic research team or community but who will contribute expertise to knowledge production processes and/or who will influence, administer or be an active user of the research results to support their decision-making. Knowledge users are often members of groups for which the research holds significance. These groups may act on (or may be affected by) the research. Knowledge users might include people who will receive care that is based on the research findings. They may occupy a range of positions, such as research funders, patients or members of the public, health system and policy decision-makers, health care providers, instructors or students at training institutions, et cetera. For example, the inclusion of patient-, healthcare delivery systems- and research funding agency-representation was identified as valuable in the process of selection and refining of pilot research and quality improvement projects in diabetes care [8]. Knowledge users are the recipients of research impacts and for this reason are the key partners and contributors of expertise in the co-production of research evidence; they are distinct from broader groups in which the research endeavour is situated.

**Fig. 1:**
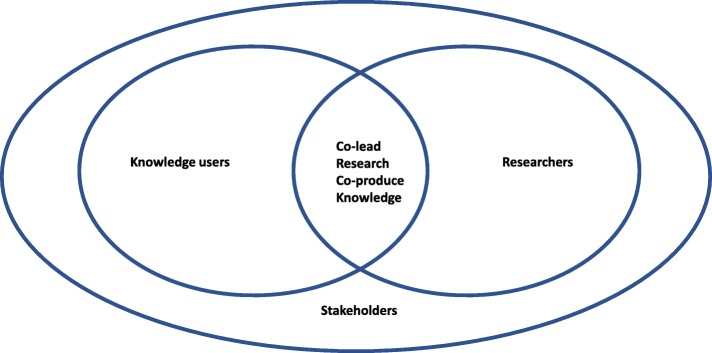
Defining interests in co-production of knowledge

The term "stakeholder" is used in many different ways: we define stakeholders to include those members of the groups, in which a research partnership of knowledge user(s) and researcher(s) is situated. Stakeholders are people or organizations who may be indirectly affected by research [9]. They may have an interest in the research and use of findings but are not anticipated to directly influence, administer and/or actively use the research results in their own decision-making. For example, in studies that investigate the primary care based management of frailty with older people members of the general population would be considered stakeholders, as those who are not anticipated to influence, administer or utilize the healthcare system for services related to frailty, [10]. Stakeholders represent individuals, groups or organizations in which a knowledge user-researcher team is situated (Fig. 1).

International work is underway to promote knowledge user engagement in health research. For example, the United States-based Patient Centred Outcomes Research Institute (PCORI) has identified the engagement of knowledge users throughout the research process to be more patient centred, useful, and trustworthy and ultimately to lead to greater use and uptake of research results by the patient and broader healthcare community [5]. Beyond identifying engagement as valuable, PCORI requires engagement in much of its funded research. PCORI works to shape the field and provides thought leadership and sets standards for engagement in research for others to reference as a model, and summarizes and synthesizes findings that relate to the field of engagement in health research – to create accessible information and opportunities to further the learning of researchers [11]. In Canada, there have been recent, similar initiatives, with the National Strategy for Patient-Oriented Research (SPOR) that aims to foster evidence-informed health care through innovation – to improve quality, accountability and accessibility [12]. Like PCORI, SPOR aims to integrate the views and values of patients into health policy and practice through research. There are many other examples of work underway to define patient and other stakeholder engagement in health research, and that also aim to promote knowledge user engagement in health research [13–16].

There is a need to conceptualize how to do health research in ways that support the engagement of those who influence, administer and/or who are active health care system users (knowledge users). That is, it is important to gain insight on the planning, implementation, and dissemination processes of research conducted from within partnerships of knowledge users and researchers to co-produce knowledge that addresses real-world needs [17]. Understanding the ways to support engagement of knowledge users in health research can lead to greater clarity in communication about and evaluation of health research conducted with knowledge user-researcher partnerships. Identification of the models and frameworks that organize concepts relevant to knowledge user engagement in health research is a valuable endeavor.

The organization of concepts in health research models and frameworks is a way to describe how to conduct research inclusive of knowledge users that leads to the co-production of knowledge. We use Walker and Avant’s (2005) definition of "concept" as a mental representation of a phenomenon, idea, action or thing, that represents observed occurrences [18]. Concepts categorize information into meaningful constructs in the form of models and frameworks to explain phenomena. There are many ways to define the terms “models” and “frameworks” and often these terms may be conceptually different and inconsistently applied. Both terms relate to the organization of concepts; for the purposes of the work presented in this paper, we use the term “frameworks” to refer to the actual, intentional organization of concepts.

Research practices that promote knowledge user engagement in health research should be guided by conceptual frameworks to focus on and aid implementation [19, 20]; currently there exists little consensus on the essential concepts of knowledge user engagement and guidance for the effective conduct of health research [14]. Our approach was to produce an evidence synthesis that combined the strengths of the comprehensive search process conducted by PCORI, with a process of critical review [21]. Our aim was to identify and then review frameworks of knowledge user engagement in health research in a systematic manner, and to describe the concepts comprising these frameworks.

## Methods

An international team, consisting of United States- and Canadian-based members, formed a collaboration and are identified here as “the team”. The team consists of an information scientist (LD); policy and practice leads (RD, SR, KW); and researchers (JJ, TN, IDG). All members of the team shared a common interest in understanding best practices in knowledge user engagement in health research, and are either acknowledged or listed as co-authors on this paper. We used an integrated knowledge translation approach meaning that there was an interactive process of knowledge exchange among team members to produce knowledge more likely to be useful to health systems’ knowledge users [1]. The team engaged in a consensus-building process that consisted of a series of regular meetings and online communications to: 1) agree upon how to identify the concepts of knowledge user engagement in health research, 2) identify existing frameworks that capture concepts of knowledge user engagement in health research, and 3) identify, synthesize and report the concepts of knowledge user engagement described in health research frameworks.

### Data source

PCORI developed the Engagement in Health Research Literature Explorer (“PCORI Explorer”) as a searchable list of articles related to engagement in research [22]. To develop the searchable list of articles, a PubMed/MEDLINE search strategy was developed and inclusion/exclusion criteria applied to confirm the final list of articles. Selected articles were then tagged across three engagement-related categories that include: Topic, Stakeholder Involvement, and Phases of Research Engagement [23]. For more information about the search strategy used to create this open-access resource, see: https://www.pcori.org/literature/engagement-literature. The PCORI Explorer is comprised of peer-reviewed articles that have been classified by AcademyHealth researchers into four article topic types: 1) Example of Engagement in Health Research, 2) Detailed Description of Engagement in Health Research, 3) Framework, Editorial, Commentary, and 4) Evidence for Engagement [22]. The search includes articles from May 1995 and for our review include those up to December 2017.

Given the combined the strengths of PCORI’s landscape review of engagement frameworks in the development of the PCORI Explorer to obtain and then classify articles related to engagement in research, we chose to focus on the article topic type Framework, Editorial, Commentary as the data source for our review. The Framework, Editorial, Commentary section includes “Manuscripts that express a theoretical view on engagement in health research, including scientific commentaries, opinion briefs, or conceptual pieces such as models or frameworks” [23].

### Procedure

**Fig. 2:**
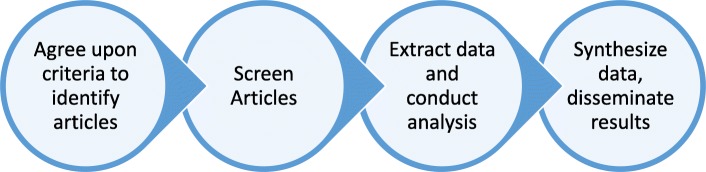
Procedure

Our study procedure was as follows: 1) determine and agree upon a set of screening criteria for articles to identify frameworks that describe and/or depict concepts of knowledge user engagement in health research; 2) screen the articles within the PCORI Explorer article topic type Framework, Editorial, Commentary to identify those that report on frameworks of knowledge user engagement in health research; 3) use a predetermined approach to abstract and conduct a content analysis of information about frameworks of knowledge user engagement in health research (demographics, concepts of knowledge user engagement reported in the framework) from the identified health research frameworks, and 4) analyze and synthesize the abstracted data and disseminate results (Fig. 2).

#### Screening criteria to identify frameworks

First, the team agreed upon how to identify the concepts of knowledge user engagement in health research, and these definitions are reported earlier: “engagement”, “knowledge user”, “stakeholder”, “concept”, and “framework”. Next, the team determined a set of screening criteria to identify articles that report on frameworks depicting concepts of knowledge user engagement in health research. There were a series of meetings to test the screening criteria with example articles, and to refine and confirm the inclusion criteria. The team reached consensus for inclusion criteria and that include: 1) the article had to present what the author of the article referred to as a framework or model of knowledge user engagement in health research; or, 2) in the absence of self-labeling as framework or model, the article had to explicitly describe and/or depict (use of figure, diagram) the organization of concepts for knowledge user engagement in health research; and 3) the framework had to be about collaborative research involving a researcher and at least one other team member who is a knowledge user (i.e., patient, caregivers, family members, healthcare providers, managers, policy makers et cetera). For screening articles, we agreed to an operational version of the definition for knowledge user engagement in health research: as an intentional arrangement that creates opportunities for knowledge users to co-lead in the processes associated with planning, conducting or disseminating research that results in co-production of knowledge [5, 6]. When an article reported on a framework that is cited in another article, the original article was obtained and screened for inclusion.

#### Screening articles

**Fig. 3:**
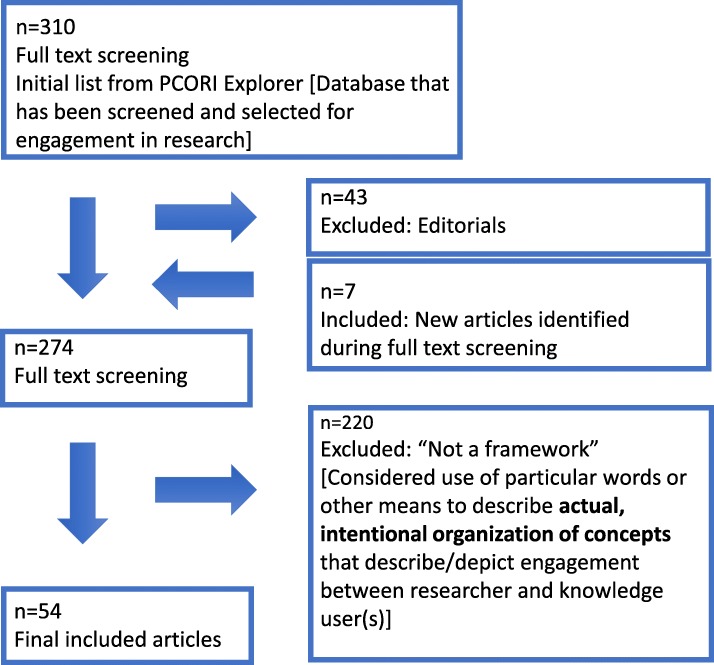
Article selection flow diagram

Next, we screened the abstracts and full text of the 310 articles, sourced from the PCORI Explorer topic section Framework, Editorial, Commentary – up to December 2017. One team member (JJ) screened articles for inclusion, with duplicate screening done independently by a second team member (TN). A third reviewer was available for discussion for conflict between the reviewers (IDG), and there were opportunities with regular meetings and shared documents for process review by the entire team (JJ, LD, RD, TN, KW, SR, IDG). As well, during the screening process, there were team meetings to discuss articles and ensure the application of the screening criteria in an agreed-upon manner. During screening an additional seven articles (external to the PCORI Explorer) were identified because they had been reported as a source of frameworks in the reviewed articles; these additional articles were also obtained and screened (Fig. 3).

#### Abstract and conduct a content analysis

Content analysis is a useful way to describe ideas and to generate or extend ways of considering organization of concepts [24]. With directed content analysis, pre-existing theory or research is used to develop the initial coding scheme [24]. During the analysis, additional codes can be developed and the initial coding scheme can be revised or refined; in this way, existing approaches to theory or research can be extended and/or refined [25]. We describe the use of a directed content analytic approach, and that involves the examination of the reported aims and concepts in articles that were identified to report the use of particular words and/or depict (with the use of figure, diagram) concepts for knowledge user engagement in health research [24, 25]:

1. Preparation, in which we decided upon the unit of analysis and the conduct of a review of each article for data. For our review, we agreed to use an iterative approach to collect data on pre-defined concepts of knowledge user engagement in health research and to inductively expand on and refine these concepts. We agreed upon and used the definition for co-production of knowledge [1]. Each time a new concept relating to knowledge user engagement was identified, data was extracted and previously identified articles reviewed for presence of the newly identified concepts;
2. Analysis of each article to identify and code data by a first reviewer (JJ) and with verification of a second reviewer (TN or IDG); the entire team (JJ, LD, RD, TN, KW, SR, IDG) was engaged in regular and ongoing discussion and review. For each article there was demographic data extracted. As well, we started with a core set of concepts based on an agreed-upon definition for co-production of knowledge to extract knowledge-user engagement concepts. For each framework we sought evidence of concepts that relate to plans for or actual conduct of knowledge user engagement that describe one or more of the following: i) development of research questions, ii) development of methodology, iii) data collection and/or tool development, iv) interpretation of study findings, v) crafting messages around the results and moving the results into practice [1]. During the data abstraction we refined definitions and identified additional concepts that related to engagement; and,
3. Reporting, to describe the analysis and findings. For our review, the aim was to identify and review frameworks of knowledge user engagement in health research in a systematic manner, and to describe the concepts comprising these frameworks. Following discussion, the entire team agreed upon final findings.

## Results

We identified 54 articles about frameworks for knowledge user engagement in health research and report on 15 concepts. A full list of the 54 articles is included (Additional file 1).

### Demographic data

The included articles depicting frameworks were published between 2000 and 2017, with none published prior to 2000 and with the majority released in 2016 (*n* = 14, 26% of the articles). The first authors of the articles were reported to be located across eight countries (Netherlands, Switzerland, Spain, Canada, Australia, United Kingdom, United States) with the highest number of first authors located in the United States (*n* = 29, 50%). Knowledge users were explicitly identified in the articles as coauthors (*n* = 8, 15%) acknowledged on the publication (*n* = 24, 44%) or not identified on the publication (*n* = 30, 55%). The number (range) of knowledge users who were reported as participating with researchers ranged from none [26] to well-over over 200 [27].

In the articles, there were many and different combinations of individual and group terms used to describe/represent knowledge users in the articles such as: patients, members of the public, stakeholders, service users, consumers, expert participants, survivors, clinicians/healthcare providers, peer support workers, mentees, decision and policy makers, et cetera. As well, terms for members of group and institutional teams were used, such as: institutional review boards, sponsors, community (includes different versions, such as rural community, community stakeholders et cetera), healthcare team (includes terms such as palliative care team), community based organizations, et cetera. The range of descriptions for knowledge users indicates a breadth of consideration for who to consider in research partnerships and how they are labeled.

### Concepts for knowledge user engagement

**Fig. 4:**
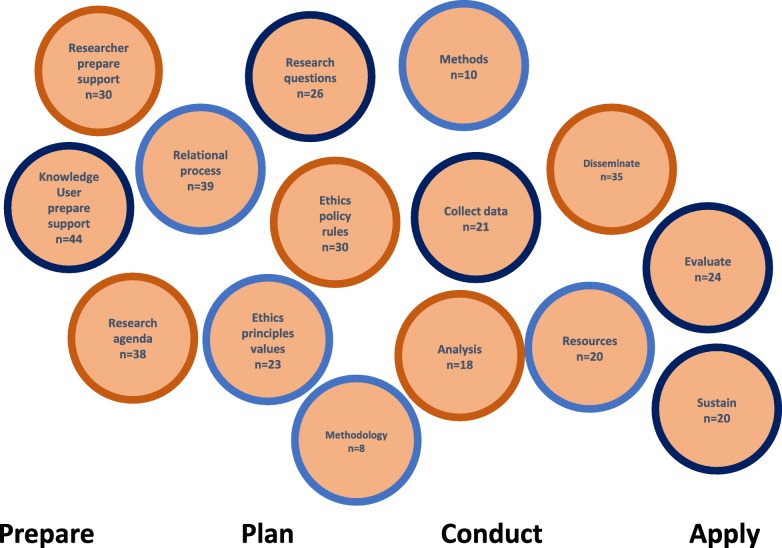
Reporting of concepts

During the content analysis of frameworks, we identified 15 concepts for knowledge user engagement reported across the 54 articles. We report these concepts with a definition and an example (Table 1). The 15 concepts are reported across four general research phases: Prepare (as a precursor to the study), Plan (the design of the study), Conduct (the study), Apply (findings of the study) and do not mean to imply an order or denote the importance of the concepts relative to one another. For example, the concept of “ethics - principles values” was most often reported in articles when describing preparation for research; however, the concept is also evident throughout other research phases in multiple articles. The average number of concepts reported in the 54 articles is *n* = 7, and range from *n* = 1 to *n* = 13 concepts. The three most commonly reported concepts include: knowledge user - prepare, support (*n* = 44), relational process (*n* = 39), research agenda (*n* = 38). The three least commonly reported concepts include: methodology (*n* = 8), methods (*n* = 10) and analysis (*n* = 18) (Fig. 4).

**Table 1:** Concepts for knowledge user engagement in health research

Concepts	Description of collaborative research process	Example
Researcher: prepare, support	Initiate/support researcher capacity/behaviour for power sharing, expertise, engagement - includes language and knowledge differences, learning (e.g. attending meetings with community groups, volunteering, and working with groups to understand knowledge user perspectives).	For example, James et al. (2011) describe how researchers were integrated into the community to learn more about community, the expertise that community members bring to research, and impacts on health disparities [28].
Knowledge user: prepare, support	Initiate/support knowledge user/community organizational capacity/behaviour for power sharing, expertise, engagement (e.g. develop resource manual, provide training in research methods).	Johnson et al. (2016) describe a study about breast cancer and how the researchers worked to prepare patients in advance of the study, so that patients felt confident in their role [29].
Relational process	Initiate and/or sustain a relational process (relationship building) between knowledge user-researcher to promote respect, reciprocity, trust and partnership synergy.	Jinks et al. (2016) conclude that inclusion of patients and the public through governance and operational levels of the research creates a culture that partnership with patients and public are an essential feature of research [30].
Research agenda	Engage in a process to define study agenda: scope, priorities, objective(s).	Dickert and Sugarman (2005) describe the inclusion of communities in planning the conduct of studies as contributing to the ethical goals of research [31].
Ethics: principles/values	Conduct knowledge user-researcher partnership work in an ethical way demonstrated by reflection on ethical concepts, and/or concern with particular values and research conducted in ways reported as meaningful, respectful, inclusive of those in the research partnership. Evidence of principled (versus policy, rules) research conduct.	In the conceptualization of implementation partnerships, Hunt et al. (2012) identify respect for community values as one of 12 guiding principles [32].
Research questions	Define research questions to identify what, specifically, the research project aims to achieve to justify the need to conduct the research (i.e. how/why was this topic chosen? What gap will it fill?).	A National Institute for Health Research (2015) reports on how working with service-user researchers in designing studies is important to keep research questions focused on concerns of those who will ultimately benefit from the research [33].
Resources	Develop funding applications/grant proposals for and/or to obtain resources (e.g. funding, time) to support knowledge user-researcher engagement.	As part of incorporating culture and diversity into translational research, Graham et al. (2016) discuss the importance of culturally specific implementation resources [34].
Ethics: policy/rules	Conduct knowledge user-research partnership work in an ethical way demonstrated by participation in an ethical application development (e.g. writing consent forms et cetera), review (e.g. research ethics board, community review) and/or development and/or use of an ethical framework (e.g. knowledge user role in the use of particular protocols, processes).	Jull et al. (2016) describe how the ethical guidance was adhered to and used to guide the conduct of the study to ensure that ethical obligations to research participants and the broader community were met and goals of the study achieved [35].
Methodology	Decide on the research methodology (approach) or report process to justify the use of the proposed methodology.	Fagan et al. (2016) describe the result of collaboration/ dialogue between the investigator and patient and family advisors about methodology as exploration of how to produce valid research and understand the potential impacts on the patient [36].
Methods	Decide upon research methods and a justification for the use of the proposed methods; selection of outcome measures.	Heaven et al. (2016) report on a study examining frailty, and how community members who were members of the study partnership were included in a process of decisions about study methods [10].
Collect data	Collect data and includes tool development.	In a study about engagement of families of children with serious acute illness, Sauers-Ford et al. (2016) describe using family feedback to adjust tools used in the collection of data [37].
Analysis	Decide about the analysis and interpretation of data (e.g. what form of analysis and how will be conducted).	In a study aimed at addressing disparities in asthma outcomes, Shelef et al. (2016) report on how preliminary analysis of the data was discussed in depth with the stakeholder engagement and national advisory core groups, and findings used to inform next steps of the study [38].
Disseminate	Identify the appropriate audience to disseminate the research findings and tailoring the message and medium to the audience to create tangible products (e.g. publication of findings, community meetings, et cetera).	Woolf et al. (2015) describe processes of engagement with the community in health equity research and that led to, among other outcomes, dissemination of findings at community meetings and newsletters [39].
Evaluate	Evaluate the research study processes.	Deverka et al. (2013) describe the evaluation of community based participatory research processes as a feature of their framework to define stakeholder engagement in comparative effectiveness research [40] .
Sustain	Maintain study benefits at a certain rate, level. That is, make deliberate efforts to sustain study intervention(s).	In describing strategies for academic and clinician engagement in community-participatory partnered research, Jones and Wells (2007) identify the support of sustainable leadership [41].

**Fig. 5:**
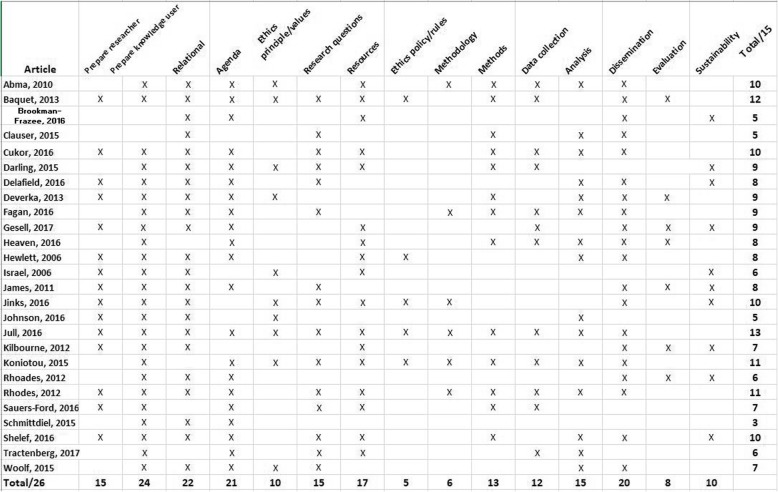
Articles describing how research was done to foster knowledge user engagement in health research: reporting across concepts (n=26)

**Fig. 6:**
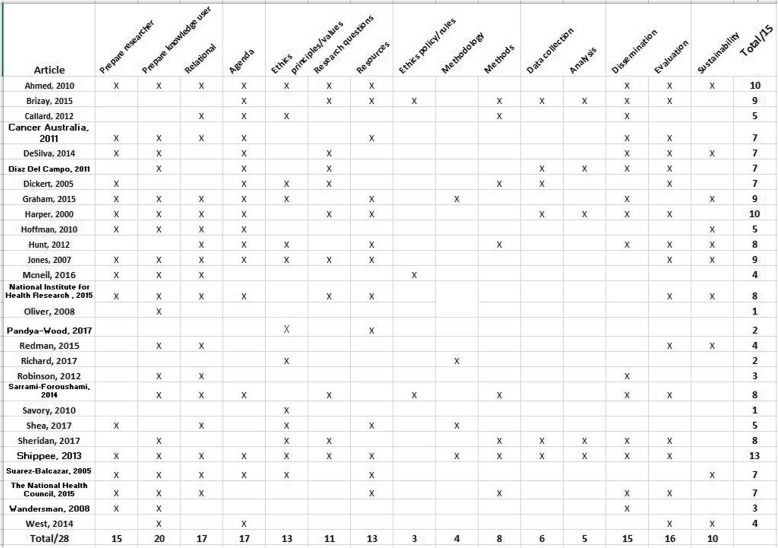
Articles describing how research should be done to foster knowledge user engagement in health research: reporting across concepts (n=28)

Further, articles can be classified in one of two categories as they either report on frameworks that describe how research *was* done or report on frameworks that are the result of considering how research *should be* done. That is, the articles were found to either 1) describe how research was done and to have reported on what had occurred in a study about knowledge user engagement in health research (*n* = 26); or, 2) describe how research should be done to foster knowledge user engagement in health research, that is, to report on a literature review, expert opinion, analysis of theory about knowledge user engagement in health research et cetera (versus actual health research conduct) (*n* = 28). The articles are organized into two framework categories and with reporting across concepts: articles describing how research was done to foster knowledge user engagement in health research (Fig. 5), and; articles describing how research should be done to foster knowledge user engagement in health research (Fig. 6). We discuss the similarities and differences between the two groups of articles.

### A comparison of frameworks that describe “How research was done” with “How research should be done”

In a comparison of the articles that report on frameworks that describe how research was done with how research should be done, those that report how research was done have a higher average number of concepts (*n* = 8) in comparison to articles that reported on how research should be done (*n* = 6). For frameworks that describe how research was done, the range of concepts reported was between *n* = 13 and *n* = 3: an article that describes collaborative research with an Indigenous community (*n* = 13) [35] and an article that describes priority setting with patients (*n* = 3) [8]. For frameworks that describe how research should be done (*n* = 28) the range of concepts reported was between *n* = 13 and *n* = 1: an article that reports on a systematic review and environmental scan of the literature about patient and service user engagement in biomedical and health services research (*n* = 13) [42] and articles reporting on patient and public involvement in translational science (*n* = 1) [43], and on the advantages and disadvantages of public involvement in health services research agenda setting (*n* = 1) [26].

Overall, the articles that report on frameworks that describe how research was done reported concepts more often. One exception was the “evaluate” concept that was reported less often in frameworks that describe how research was done (38%) relative to frameworks that describe how research should be done (57%). The greatest differences in frequency of concept reporting was found for the concept of “resources” (how research was done = 58% versus how research should be done =18%) and the smallest difference was found for the concept “sustain” (how research was done = 38% versus how research should be done = 36%) (Table 2).

**Table 2:** Frequency of concept reporting for articles that report how research was done in relation to how research should be done

Concepts	How research was done (*n* = 26)	How research should be done (*n* = 28)
Researcher - prepare, support	15/26 (57%)	15/28 (53%)
Knowledge user - prepare, support	24/26 (92%)	20/28 (71%)
Relational process	22/26 (85%)	17/28 (61%)
Research agenda	21/26 (81%)	17/28 (61%)
Ethics – principles, values	10/26 (38%)	13/28 (46%)
Research questions	15/26 (58%)	11/28 (39%)
Resources	15/26 (58%)	5/28 (18%)
Ethics: policy/rules	17/26 (65%)	13/28 (45%)
Methodology	5/26 (19%)	3/28 (11%)
Methods	6/26 (23%)	4/28 (14%)
Collect data	13/26 (50%)	8/28 (29%)
Analysis	12/26 (46%)	6/28 (21%)
Disseminate	20/26 (77%)	15/28 (64%)
Evaluate	8/26 (31%)	16/28 (57%)
Sustain	10/26 (38%)	10/28 (36%)

## Discussion

The aim of our work was to identify and review frameworks of knowledge user engagement in health research in a systematic manner, and to describe the concepts comprising these frameworks. To do this work, we identified, reviewed and synthesized 54 articles that reported on frameworks meeting our criteria for describing and/or depicting knowledge user engagement in health research. Our analysis identified 15 concepts related to knowledge user engagement in health research. The variation in the reported concepts between the frameworks means that it is difficult to conclude what and in which order might be best for knowledge user engagement in health research. Research teams should think about engagement with the concepts as being fluid rather than strictly required, as there is a lack of evidence at this time for the benefits of engaging knowledge users across each of the 15 identified concepts.

Understanding the concepts of knowledge user engagement in health research may help researchers and knowledge user partners to identify and operationalize engagement in health research, and in ways that account for the nuanced characteristics and preferences of particular group(s) of people. For knowledge users there are potentially many opportunities and ways to engage or be engaged in areas of research that may typically be the domain of researchers alone. There is a need to understand best practice in research engagement [44]: encouraging dialogue and debate between knowledge users and researchers about the concepts of knowledge user engagement in health research on a situational basis may contribute to processes of engagement in health research.

**Fig. 7:**
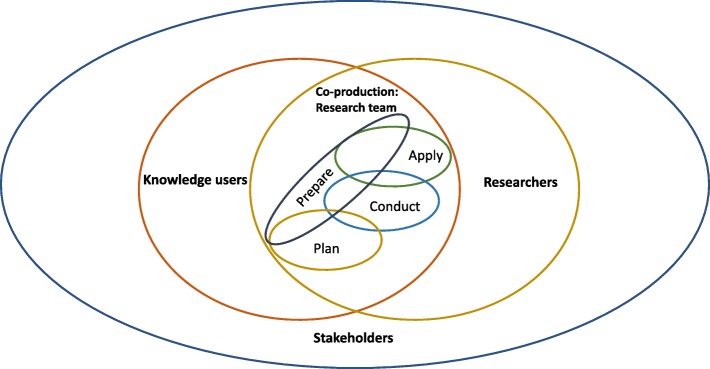
Meta-framework, knowledge user engagement in collaborative research

We propose that it is reasonable to suggest that knowledge users and researchers discuss and negotiate the research process to determine who should be meaningfully engaged and in what ways. It is important to maximize the potential to advance research project objectives to reflect the needs and aims of knowledge user partners to ensure that the outcomes are useful and impactful. As well, we propose that a form of partnered negotiation takes place at the start and throughout the research study. A process of partnered negotiation is important for considering and possibly utilizing concepts throughout the phases of research, in order to foster successful knowledge user engagement. We depict these relationships in a met-framework (Fig. 7).

In our work, we have identified the need to think about knowledge user engagement in health research as an arrangement that creates ongoing opportunities for engagement by knowledge users throughout the research process. While the promotion of full engagement of knowledge users throughout the research process is described in the literature as an ideal [5, 6], dictating when and how knowledge users and researchers need to be involved is not helpful nor is it realistic. The *equal* involvement of knowledge users and researchers throughout the research process may vary and a focus on equity may instead be more realistic for knowledge user engagement. That is, while the contributions by knowledge users and researchers may differ or vary at times, the effort and impact is similar – sharing equally is not as important as considering the quality of engagement in research processes. In our work, studies were found to report on many types of individual and collective knowledge users, and we propose that engagement with knowledge users is a form of partnered negotiation. Every research partnership is unique, as it consists of individuals (knowledge users and researchers) who, themselves, understand their unique expertise, needs and context.

The use of frameworks can guide the exploration of how to operationalize measures of impact of knowledge user engagement. The findings of our study are reinforced by the literature; there are a broad range of ways in which knowledge users might be engaged in health research and little evidence for the impact of this engagement [45]. As well, there has been an important - and dominant - focus on patient and lay involvement in health research [46]. Our work shows that engagement of knowledge users in health research is more complex than bringing in one form of knowledge user: the pursuit of knowledge user engagement in health research means that there are teams consisting of researchers and knowledge users that span health care systems. The evaluation of knowledge user engagement in health research is identified as an area in need of development [17, 44–47]. To build understandings and unravel the complexity of knowledge user engagement in health research, it is imperative that research studies utilize guiding frameworks. For example, PCORI (a major research funder in the United States) encourages that those conducting funded projects evaluate the engagement of knowledge users (defined as patients and other stakeholders, such as clinicians, purchasers, payers et cetera) with the PCORI Engagement Rubric [5, 48, 49]. The aim of the evaluation is to develop knowledge about engagement practices with knowledge users. Our finding of lower reporting of the “evaluate” concept suggests that there may be some barriers to the actual conduct of evaluation in frameworks of knowledge user engagement in health research. To advance the field of knowledge user engagement in health research, future efforts should focus on evaluation.

In addition to the evaluation of engagement practices with knowledge users, we propose that studies need to go beyond reporting on the framework used for knowledge user engagement in health research, and present analysis about the use of frameworks that guide research. Many studies report on the use of frameworks to guide knowledge user engagement in health research; however, there are few examples of studies that systematically evaluate these guiding frameworks for their use in guiding teams consisting of knowledge users and researchers [11, 50]. Studies that evaluate the guiding frameworks for knowledge user engagement in health research are important for understanding how to engage people at different points in the study, and in what ways. Here we have reported on a range of included studies that show the complexity and variability of knowledge user engagement, across research stages and concepts. To advance the field of knowledge user engagement in health research, it is imperative that there is evaluation of the frameworks guiding knowledge user engagement.

### Limitations and strengths

A key limitation of our study is that we may have included or excluded articles inappropriately due to the conceptually different and inconsistently used range of terms and descriptions for models and frameworks. For the purposes of this review, and in an attempt to mitigate issues with terminology, we agreed upon an operational definition to aid in the identification of models and frameworks. In addition, our study used a source of articles designed to capture the general literature about engagement in health research, rather than a search designed and conducted specifically for our purposes.

Strengths of our review include the use of the PCORI Explorer to identify articles, and the involvement of an international team with the use of a consensus-building process. The PCORI Explorer is a repository of peer-reviewed articles focused on guidance in the field of collaborative research conduct and that includes frameworks, conceptual models, and editorials to express a theoretical view on engagement in health research. Those who oversee the PCORI Explorer repository also included articles that are recommended by PCORI staff, or journals identified as highly relevant despite not yet being indexed in PubMed [23]. Additionally, an international team with experience in conceptualizing the engagement of knowledge users in collaborative research across a range of settings included those with expertise in the use of the PCORI Explorer (LA, RD, SR, KW) and use and/or development of knowledge translation frameworks (JJ, TN, IDJ).

## Conclusions

The aim of our work was to identify and review frameworks of knowledge user engagement in health research in a systematic manner, and to describe the concepts comprising these frameworks. We identify 15 concepts of knowledge user engagement in health research, and suggest that to advance the field of knowledge user engagement in health research there is a need to focus on evaluation. Given the variation in the number of concepts reported across the included frameworks it is difficult to say what and in which order concepts are best for collaborative research. Further work to develop understandings of knowledge user engagement in health research is encouraged.

## Supplementary information

**Additional file 1.** List of included articles.

## Data Availability

Supporting data may be found at the PCORI Explorer https://www.pcori.org/literature/engagement-literature repository, and the datasets used and/or analysed during the current study are available from the corresponding author on reasonable request.
