# Supplementary material for: A review and synthesis of frameworks for engagement in health research to identify concepts of knowledge user engagement
Source: BMC Med Res Methodol. 2019 Nov 21;19:211. doi: 10.1186/s12874-019-0838-1 (PMC6869315; doi:10.1186/s12874-019-0838-1)
Supplement: Supplementary file 1 — Additional file 1. List of included articles. [file 12874_2019_838_MOESM1_ESM.docx]

Additional file 1: List of included articles

1. Abma TA, Broerse JE. Patient participation as dialogue: setting research agendas. Health Expect. 2010 Jun; 13 (2):160-73.

Objective: To develop a methodology for health research agenda setting processes grounded in the notion of participation as dialogue (develop and test the Dialogue model).

1. Ahmed SM, Palermo AG. Community engagement in research: frameworks for education and peer review. Am J Public Health. 2010 Aug; 100 (8):1380-7.

Objective: The National Institutes of Health Director’s Council of Public Representatives developed a community engagement framework that includes values, strategies to operationalize each value, and potential outcomes of their use, as well as a peer- review framework for evaluating research that engages communities.

1. Baquet, CR, et al. Rural community-academic partnership model for community engagement and partnered research. Progress in Community Health Partnerships. 2013 7(3):281-90.

Objective: The primary aim was to develop a sustainable community–academic partnership that addressed health and social issues on the rural Eastern Shore.

1. Brizay U et al. Community-academic partnerships in HIV-related research: a systematic literature review of theory and practice. Journal of the International AIDS Society. 2015 Jan 27; 18:19354.

Objective: To review the existing terms and definitions regarding community-academic partnerships and assess how studies are implementing these in relation to conceptual definitions.

1. Brookman-Frazee L et al. Characterizing the Use of Research-Community Partnerships in Studies of Evidence-Based Interventions in Children's Community Services. Administration and Policy in Mental Health and Mental Health Services Research. 2016 Jan; 43(1):93-104.

Objective: This study characterized the use of research community partnerships to tailor evidence- based intervention, training, and implementation models for delivery across different childhood problems and service contexts using a survey completed by project principal investigators and community partners.

1. Callard F et al. Close to the bench as well as at the bedside: involving service users in all phases of translational research. Health Expectations. 2012 Dec; 15(4):389-400. Objective: The paper aims to develop a model of translational research in which service user and other stakeholder involvement are central to each phase.
2. Cancer Australia, 2011. National Health and Medical Research Council (NHMRC)/Consumers' Health Forum of Australia A Model Framework for Consumer and Community Participation in Health and Medical Research. Commonwealth of Australia. 2004.

Objective: The National Framework for Consumer Involvement in Cancer Control has been developed by Cancer Australia in partnership with Cancer Voices Australia to enhance meaningful consumer involvement at all levels of cancer control in order to improve outcomes and experiences for people affected by cancer.

1. Clauser SB et al. Patient Centeredness and Engagement in Quality-of-Care Oncology Research. Journal of Oncology Practice, 2015 11(3): 176-9.

Objective: The article describes advancing the goal of partnership with diverse interdisciplinary research teams who demonstrate a strong commitment to the inclusion and engagement of patients and stakeholders as they work to develop high-quality cancer care delivery systems.

1. Cukor D et al. Patient and Other Stakeholder Engagement in Patient-Centered Outcomes Research Institute Funded Studies of Patients with Kidney Diseases. Clinical Journal of the American Society of Nephrology. 2016 Sep 7; 11(9):1703-12. Objective: To explore the experiences among PCORI funded projects that target patients with kidney disease to gain an understanding of how meaningful patient and stakeholder engagement is being operationalized by the research team.
2. Darling M et al. Practical Tips for Establishing Partnerships with Academic Researchers: A Resource Guide for Community-Based Organizations. Progress in Community Health Partnerships. 2015 Summer; 9(2):203-12.

Objective: To create a resource for CBOs that describes how an organization can prepare for and participate in CBPR.

1. De Silva MJ et al. Theory of Change: a theory-driven approach to enhance the Medical Research Council's framework for complex interventions. Trials. 2014 Jul 5; 15:267.

Objective: To propose a theory-driven approach to the design and evaluation of complex interventions by adapting and integrating a programmatic design and evaluation tool, Theory of Change, into the Medical Research Council framework for complex interventions.

1. Delafield et al. A Community-Based Participatory Research Guided Model for the Dissemination of Evidence-Based Interventions. Progress in Community Health Partnerships: Research, Education, and Action. 2016 10(4):585-595.

Objective: The community-to-community mentoring (CCM) model described in this paper extends the application of CBPR values and principles used in intervention development to intervention dissemination.

1. National Institute for Health Research. Going the Extra Mile: Improving the nation’s health and wellbeing through public involvement in research. The final report and recommendations to the Director General Research and Development/Chief Medical Officer Department of Health of the ‘Breaking Boundaries’ strategic review of public involvement in the National Institute for Health Research. 2015

Objective: To present results on a strategic review documented in the principles section of this chapter that offer a starting point from which to evolve and improve public involvement in research.

1. Deverka PA et al. Stakeholder participation in comparative effectiveness research: defining a framework for effective engagement. Journal of Comparative Effectiveness Research. 2012 Mar; 1(2):181-194.

Objective: Stakeholder engagement is fundamental to comparative effectiveness research (CER), but lacks consistent terminology. This paper aims to define stakeholder engagement and present a conceptual model for involving stakeholders in CER.

1. Díaz Del Campo P et al. A strategy for patient involvement in clinical practice guidelines: methodological approaches. BMJ Quality and Safety. 2011 Sep; 20(9):779-84.

Objective: To present a strategy for patient involvement that includes both robust patient consultation and participation. This strategy is based on the authors’ experience in the development of four clinical practice guidelines (CPGs) included in the Spanish National CPGs Development Program, which deemed it a valuable nation-wide patient involvement initiative.

1. Dickert N & Sugarman J. Ethical goals of community consultation in research. American Journal of Public Health. 2005 Jul; 95(7):1123-7.

Objective: To propose 4 ethical goals that give investigators, sponsors, institutional review boards, and communities a framework for evaluating community consultation processes.

1. Fagan MB et al. Implementing a pragmatic framework for authentic patient-researcher partnerships in clinical research. Journal of Comparative Effectiveness Research. 2016 May; 5(3):297-308.

Objective: The purpose of this paper is to describe a framework of collaborative engagement between investigators and patient family advisors and to identify determinants that enable this research approach at academic medical centres.

1. Gesell SB et al. Methods guiding stakeholder engagement in planning a pragmatic study on changing stroke systems of care. Journal of Clinical and Translational Science. 2017 Apr; 1(2):121-128.

Objective: The purpose of this article is to address gaps in the literature on stakeholder engagement in comparative effectiveness research by presenting the model that guides our stakeholder engagement efforts, and sharing the methods we used throughout the pre-award and post-award study design periods to inform best practice methods for engaging stakeholders in large pragmatic trials.

1. Graham PW et al. What is the role of culture, diversity, and community engagement in transdisciplinary translational science? Translational Behavioral Medicine. 2016 Mar; 6(1):115-24.

Objective: To present a conceptual framework focused on the seamless incorporation of culture and diversity through- out the various stages of the translational science process. Our comprehensive model for incorporating culture and diversity into translational re- search provides a basis for further discussion and translational science development.

1. Harper GW & Salina DD. Building Collaborative Partnerships to Improve Community-Based HIV Prevention Research. Journal of Prevention & Intervention in the Community. 2008, 19 (1): 2000.

Objective: The article presents a 6 stage conceptual model of effective university-CBO partnerships, with emphasis on HIV prevention research.

1. Heaven A et al. Keeping it credible in cohort multiple Randomized Controlled Trials: the Community Ageing Research 75 (CARE 75) study model of patient and public involvement and engagement. Research Involvement and Engagement. 2016; 2(30). DOI: 10.1186/s40900-016-0044-9

Objective: To create a structure to enable meaningful, sustainable public involvement within the cohort multiple randomized control trial framework. We have established a core reference group of four key individuals with extensive links to other relevant local community structures and individuals.

1. Hewlett S et al. Patients and professionals as research partners: challenges, practicalities, and benefits. Arthritis & Rheumatology. 2006 Aug 15; 55(4):676-80.

Objective: This article reports the combined experiences of researchers and patients who are collaborating in rheumatology research, and the working model that has evolved. The article provides one example of a practical model for collaboration based on experience.

1. Hoffman A et al. How best to engage patients, doctors, and other stakeholders in designing comparative effectiveness studies. Health Affairs (Millwood). 2010 Oct; 29(10):1834-41.

Objective: Through a qualitative assessment of case studies, the authors identify five key principles for the effective engagement of a broad coalition of participants in research intended to improve health care and control costs.

1. Hunt JB et al. Partnership for implementation of evidence-based mental health practices in rural federally qualified health centers: theory and methods. Progress in Community Health Partnerships. 2012 6(3): 389-98.

Objective: To present a conceptual model that integrates seven separate frameworks: 1) Jones and Wells’ Evidence-Based Community Partnership Model, 2) Kitson’s Promoting Action on Research Implementation in Health Services (PARiHS) implementation framework, 3) Sackett’s definition of evidence-based medicine, 4) Glisson’s organizational social context model, 5) Rubenstein’s Evidence-Based Quality Improvement (EBQI) facilitation process, 6) Glasgow’s RE-AIM evaluation approach, and 7) Naylor’s concept of shared decision making.

1. Israel BA et al. Challenges and facilitating factors in sustaining community-based participatory research partnerships: lessons learned from the Detroit, New York City and Seattle Urban Research Centers. Journal of Urban Health. 2006 Nov;83(6):1022-Objective: The purpose of this article is to examine the experiences and lessons learned from three Urban Research Centers in Detroit, New York City, and Seattle, initially established in 1995 with core support from the Centers for Disease Control and Prevention.
2. James S et al. Community ACTION boards: an innovative model for effective community-academic research partnerships. Progress in Community Health Partnerships. 2011 Winter;5(4):399-404.

Objective: The ECHHO board sought to identify key strategies and tools to build and employ a partnership model, and to disseminate lessons learned to other community–academic partnerships.

1. Jinks C et al. Patient and public involvement in primary care research - an example of ensuring its sustainability. Research Involvement and Engagement. 2016; 2(1). Objective: This paper focuses on the issues of sustainability, the importance of institutional leadership and the creation of a robust infrastructure in order to achieve long-term and wide-ranging patient and public involvement in research strategy and programmes.
2. Johnson D et al. The patient voice in research - evolution of a role. Research Involvement and Engagement. 2016; 2(6).

Objective: We use our experience to share how the patient voice can affect the execution of a research study and to provide a model for meaningfully engaging patients in research.

1. Jones L & Wells K. Strategies for academic and clinician engagement in community-participatory partnered research. JAMA. 2007 Jan 24; 297(4):407-10.

Objective: To present strategies to build effective partnerships that facilitate integration of perspectives of diverse community and academic members in community-partnered participatory research.

1. Jull J et al. Development of a collaborative research framework: the example of a study conducted by and with a First Nations, Inuit and Métis women's community and their research partners. ACME: An International Journal for Critical Geographies (University of British Columbia) [S.1.], Oct. 2016. ISSN 1492-9732.

Objective: The purpose of this paper is to describe the development of a collaborative framework defined by community members and their research partners as ethical, useful and relevant.

1. Kilbourne AM et al. Public-academic partnerships: evidence-based implementation: the role of sustained community-based practice and research partnerships. Psychiatric Services. 2012 Mar; 63(3):205-7.

Objective: To compare the enhanced framework with the center for disease control’s standard procedures in enhancing fidelity and improving health outcomes for patients randomly assigned to the two conditions.

1. Koniotou M et al. Involving older people in a multi-centre randomised trial of a complex intervention in pre-hospital emergency care: implementation of a collaborative model. Trials. 2015 Jul 10; 16:298.

Objective: We implemented a model to involve service users in a multi-centre randomised controlled trial in pre-hospital emergency care. We used the generic Standard Operating Procedure from our Clinical Trials Unit as the basis for creating a model to fit the context and population of the SAFER 2 trial.

1. Mcneil H et al. Engaging older adults in healthcare research and planning: a realist synthesis. Research Involvement and Engagement. 2016; 2(1).

Objective: This project aimed to develop an understanding of engagement of older adults and their caregivers in healthcare research and planning. A five-stage engagement framework emerged from this study that can be used to guide engagement efforts.

1. Oliver SR et al. A multidimensional conceptual framework for analysing public involvement in health services research. Health Expectations. 2008 Mar;11(1):72-84. Objective: In 1999, the Health Technology Assessment (HTA) programme commissioned primary research and a literature review about the advantages and disadvantages of different methods of public involvement in identifying and prioritizing topics for research. The aim of this paper is to reflect on the development and utility of the conceptual framework we developed for that review.
2. Pandya-Wood R et al. A framework for public involvement at the design stage of NHS health and social care research: time to develop ethically conscious standards. Research Involvement and Engagement. 2017; 3(1).

Objective: We have observed ten areas where ethical issues may arise because of the actions researchers may or may not take and which might consequently have a negative impact. Therefore, we have used these observations to develop a “framework” to help researchers and the public work together at the early design stage in ways that are ethical.

1. Redman S et al. The SPIRIT Action Framework: A structured approach to selecting and testing strategies to increase the use of research in policy. Social Science and Medicine. 2015 Jul; 136-137:147-55.

Objective: This paper describes the development of the SPIRIT Action Framework. The framework provides a pragmatic basis to develop and test interventions.

1. Rhoades BL et al. The role of a state-level prevention support system in promoting high-quality implementation and sustainability of evidence-based programs. American Journal of Community Psychology. 2012 Dec; 50(3-4):386-401.

Objective: In this article we expand on the Interactive Systems Framework for Dissemination and Implementation model by presenting funders and policy-makers as active and engaged stakeholders, and demonstrate how a state-level prevention support system has used empirical evidence to inform general and program-specific capacity- building and support interactions among researchers, funders, and practitioners in Pennsylvania.

1. Rhodes SD et al. Using community-based participatory research (CBPR) to develop a community-level HIV prevention intervention for Latinas: a local response to a global challenge. Women’s Health Issues. 2012 May-Jun; 22(3):e293-301.

Objective: The purpose of this project was to develop an intervention that builds on existing community strengths to promote sexual health among immigrant Latinas.

1. Richard L et al. Advancing engagement methods for trials: the CORE study relational model of engagement for a stepped wedge cluster randomized controlled trial of experience-based co-design for people living with severe mental illnesses. Trials. 2017 April 8; 18(1):169.

Objective: This article describes an innovative methodological approach for the development and application of a relational model of engagement in a stepped wedge designed cluster randomised controlled trial (RCT), the CORE study, that will test if an experience-based co-design intervention improves psychosocial recovery for people affected by severe mental illness

1. Robinson L et al. Professionals and the public: power or partnership in health research? Journal of Evaluation in Clinical Practice. 2012 Apr; 18(2):276-82. Objective: To present a conceptual model for evaluating and reporting the impact of public involvement in health research.
2. Sarrami-Foroushani P et al. Implementing strategies in consumer and community engagement in health care: results of a large-scale, scoping meta-review. BMC Health Services Research. 2014 Sep 18;14:402.

Objective: Despite the long history of consumer and community engagement (CCE) research and practice, there is no consensus on the best strategies for CCE. In this paper, we identify various dimensions of CCE-related strategies and offer a practical model to assist policy-makers, practitioners and researchers.

1. Sauers-Ford HS et al. Strategies to engage stakeholders in research to improve acute care delivery. Journal of Hospital Medicine. 2016 Feb; 11(2):123-5.

Objective: In this Perspective, we describe our experiences with family engagement using a novel approach of serial, focused, short-term engagement of stakeholders.

1. Savory C. Patient and public involvement in translative healthcare research. Clinical Governance: An International Journal. 2010; 15(3): 191-199.

Objective: This paper sets out how various Patient and Public Involvement strategies can be united within a distinct framework by drawing on perspectives and practices for managing technological innovation, from sectors other than healthcare.

1. Schmittdiel JA et al. Methods for engaging stakeholders in comparative effectiveness research: a patient-centered approach to improving diabetes care. Healthcare (Amsterdam, Netherlands). 2015 Jun;3(2):80-8.

Objective: We developed a 5-step approach to engaging a diverse set of stakeholders to help us identify and prioritize clinical effectiveness research questions that are most relevant to diabetes care and prevention, and guide the enhancement of the infrastructure and sustainability of the DataLink.

1. Shea CM et al. Researcher readiness for participating in community-engaged dissemination and implementation research: a conceptual framework of core competencies. Translational Behavioral Medicine. 2017 Mar 24.

Objective: To propose a conceptual framework that identifies detailed competencies for researchers participating in community-engaged dissemination and implementation research and maps these competencies to domains.

1. Shelef DQ et al. Using stakeholder engagement to develop a patient-centered pediatric asthma intervention. Journal of Allergy and Clinical Immunology. 2016 Dec; 138(6):1512-1517.

Objective: To describe the effect of stakeholder engagement on the design of a randomized trial of an intervention designed to improve child asthma outcomes by reducing parental stress.

1. Sheridan S et al. The PCORI Engagement Rubric: Promising Practices for Partnering in Research. The Annals of Family Medicine. 2017 Mar; 15(2):165-170.

Objective: To address the need for guidance on creating meaningful stakeholder partnerships in patient-centered clinical comparative effectiveness research, the Patient-Centered Outcomes Research Institute (PCORI) developed the PCORI Engagement Rubric (Rubric).

1. Shippee ND et al. Patient and service user engagement in research: a systematic review and synthesized framework. Health Expectations. 2015 Oct; 18(5):1151-66. Objective: This paper utilizes a systematic review and environmental scan to derive an evidence-based framework for patient and service user engagement.
2. Suarez-Balcazar Y et al. An interactive and contextual model of community-university collaborations for research and action. Health Education & Behavior. 2005 Feb; 32(1):84-101. PubMed PMID: 15642756.

Objective: This article proposes an interactive and contextual model for developing and sustaining community-university partnerships.

1. The National Health Council and Genetic Alliance. Dialogue / Advancing Meaningful Patient Engagement in Research, Development, and Review of Drugs. Sep 2015.

Objective: The findings articulated in this report are reflective of the key themes discussed at the Dialogue and organized to show how these actions pave the way for longer-term strategies.

1. Tractenberg RE et al. Maintaining primacy of the patient perspective in the development of patient-centered patient reported outcomes. 2017 PLoS ONE 12(3): e0171114.

Objective: To describe and demonstrate a new model of developing patient reported outcomes (PROs) that are patient-centred, and to test the hypothesis that following this model would qualitatively different PROs than if the typical PRO development model were followed.

1. Wandersman A et al. Bridging the gap between prevention research and practice: the interactive systems framework for dissemination and implementation. American Journal of Community Psychology. 2008 41 (3-4); 171-81.

Objective: The article presents the Interactive Systems Framework for Dissemination and Implementation (ISF) that uses aspects of research to practice models and of community‐centered models.

1. West JF. Public health program planning logic model for community engaged type 2 diabetes management and prevention. Evaluation and Program Planning. 2014 Feb; 42:43-9.

Objective: A conceptual model emphasizing action-oriented micro-level community engagement is needed to complement the Community Guide and serve as the basis for testing and evaluation of these kinds of interventions.

1. Woolf SH et al. Translating evidence into population health improvement: strategies and barriers. Annual Review of Public Health. 2015 Mar 18; 36:463-82.

Objective: We share examples from our work, including the engagement of patients in a study of cancer screening decisions and the engagement of inner-city residents in addressing social determinants of health.
